# A Systematic Review and Meta-Analysis of the Risk of Stillbirth, Perinatal and Neonatal Mortality in Immigrant Women

**DOI:** 10.3389/ijph.2022.1604479

**Published:** 2022-05-18

**Authors:** Samira Behboudi-Gandevani, Razieh Bidhendi-Yarandi, Mohammad Hossein Panahi, Abbas Mardani, Ingjerd Gåre Kymre, Piret Paal, Mojtaba Vaismoradi

**Affiliations:** ^1^ Faculty of Nursing and Health Sciences, Nord University, Bodø, Norway; ^2^ Department of Biostatistics, University of Social Welfare and Rehabilitation Sciences, Tehran, Iran; ^3^ Department of Epidemiology, School of Public Health and Safety, Shahid Beheshti University of Medical Sciences, Tehran, Iran; ^4^ Nursing Care Research Center, Department of Medical Surgical Nursing, School of Nursing and Midwifery, Iran University of Medical Sciences, Tehran, Iran; ^5^ Institute of Nursing Science and Practice, Paracelsus Medical University, Salzburg, Austria

**Keywords:** meta-analysis, immigration, perinatal mortality, neonatal mortality, stillbirth

## Abstract

**Objectives:** This study aimed to investigate the risk of stillbirth, perinatal and neonatal mortality in immigrant women compared to native-origin women in host countries.

**Methods:** A systematic literature review and meta-analysis was conducted. Relevant studies were identified using a thorough literature search and their quality was appraised. The analysis of heterogeneous data was carried out using the random effects model and publication bias was assessed using the Harbord-test. Also, the pooled odds ratio of events was calculated through the DerSimonian and Laird, and inverse variance methods.

**Results:** In the search process 45 studies were retrieved consisting of 8,419,435 immigrant women and 40,113,869 native-origin women. The risk of stillbirth (Pooled OR = 1.35, 95% CI = 1.22–1.50), perinatal mortality (Pooled OR = 1.50, 95% CI = 1.35–1.68), and neonatal mortality (Pooled OR = 1.09, 95% CI = 1.00–1.19) in the immigrant women were significantly higher than the native-origin women in host countries. According to the sensitivity analyses, all results were highly consistent with the main data analysis results.

**Conclusion:** The immigrant women compared to the native-origin women had the higher risks of stillbirth, perinatal and neonatal mortality. Healthcare providers and policy makers should improve the provision of maternal and neonatal healthcare for the immigrant population.

## Introduction

The number of immigrant people across the globe has increased over the past decades and now is at an all-time high [1]. The impact of immigration on people health is far-reaching as immigrants are particularly vulnerable to health inequalities [2]. The immigrant population mostly consists of women in the childbearing age that experience the negative effects of immigration on their maternal and neonatal health [3].

Perinatal mortality has been defined as the number of fetal and early neonatal deaths for every 1,000 births. Stillbirth refers to a baby born dead after the 28th week of gestation and neonatal mortality is considered death in the first 28 days of life. As important indicators of the quality of antenatal, obstetric, and perinatal care, they reflect the overall health status in the community [4]. Also, they are linked to the effective maternal and neonatal care throughout the continuum of care initiated from the pregestational period until postpartum [5–7].

Studies on the risk of perinatal mortality among the newborns of immigrant women have reported controversial results. Some studies show that perinatal mortality among these newborns generally occur more frequently than the newborns of women in host countries [8–20]. Other studies show that immigrant women have the same or even less perinatal mortality than native-origin women [4, 21–26]. Given the lack of conclusive evidence, this systemic review and meta-analysis aimed to investigate the risk stillbirth, and perinatal and neonatal mortality in immigrant women compared to native-origin women in host countries.

## Methods

The systematic review was conducted in accordance with the Cochrane methodology for systematic review and meta-analysis. The whole review process was informed by the Preferred Reporting Items for Systematic Reviews and Meta-Analyses (PRISMA) [27]. The following PICO (Population, Intervention or exposure, Comparison, Outcome) elements were applied as inclusion criteria to this systematic review: P: all women with the history of pregnancy and their neonates; I: international immigration; C: native-origin women; O: perinatal death, still birth, and neonatal mortality.

### Eligibility Criteria

Analytic observational studies with cross-sectional, case-control, and cohort designs that addressed the prevalence or risk of at least one of stillbirth, perinatal, and neonatal mortality incidents in immigrant women were considered. Other inclusion criteria were studies published in English language and should present data on immigrant women who crossed international borders compared to native-origin women. Also, they should report the number or prevalence incident of the outcomes of interest in both groups. Movement of women internally within national boundaries led to the exclusion of studies. Also, non-original studies including reviews, commentaries, editorials, letters, meeting abstracts, case reports, and conference proceedings were excluded, because they did not provide accurate and clear data on research variables. Duplicated studies, those with a focus on specific minor populations such as adolescents, and with incomplete data were excluded. No restrictions were applied based on the immigration origin, status, or length of time passed in the host country.

### Search Strategy

A thorough literature search was carried out on the databases of PubMed (including MEDLINE), Web of Science, and Scopus until September 2020. To maximize the identification of eligible studies, a manual search in the references lists of selected studies and relevant reviews was performed. Search keywords consisting of free-text and MeSH terms were combined using the Boolean method and were utilized to conduct the search: (migration OR immigration OR migrant OR immigrant OR emigrant OR asylum seeker OR asylum seeking OR asylum OR refugee) AND (stillbirth OR still birth OR stillborn OR fetal death OR perinatal mortality OR perinatal death OR neonatal mortality OR neonatal death OR adverse pregnancy outcomes OR pregnancy outcomes OR pregnancy complications OR adverse neonatal outcome).

### Study Selection and Data Extraction

The screening of titles, abstracts, and full texts of studies according to eligibility criteria was performed independently by two authors. A third reviewer was consulted in case of disagreement between two data extractors. Discrepancy was resolved by consensus. The following data were extracted from eligible studies and were exported to a table: design; country; publication year; study period; sample size; population characteristics including age and body mass index (BMI); outcome measurements including the number and prevalence of outcomes of interest. Both raw (unadjusted) and adjusted odd ratio/relative risk/hazard ratio were recorded. For the prevention of extraction errors, the original studies’ data and those data used in the meta-analysis were compared together.

### Terms Definition

The immigrant population has been defined as “any person moving across an international border, regardless of the person’s legal status; whether it was voluntary or involuntary and what causes for the movement were; or what the length of stay was” [28]. It constitutes a heterogeneous group including refugees, asylum seekers, illegal and undocumented immigrants, economic and transient immigrants.

The native-origin population has been defined as any person who has two parents born in the host country. Perinatal mortality is defined as intrauterine death at or after 22 weeks of gestational age or any early neonatal death occurring within 7 days after birth [29]. Stillbirth is fetal death at ≥ 28 weeks of gestation or weight ≥1,000 g. If both these criteria are unknown, crown-heel length ≥35 cm is considered [30]. Neonatal mortality is defined as the death of a live born infant, regardless of gestational age at birth, within the first 28 completed days of life [31].

### Quality Appraisal

Selected studies were critically appraised in terms of the methodological structure and presentation of results using the modified Newcastle–Ottawa Quality Assessment Scale [32]. Two authors who were blind to the study’s author and institution, and the journal’s title evaluated the quality of each study independently. Studies with scores above 6 were considered high quality, 4–6 moderate quality, and less than 4 low quality.

### Patient and Public Involvement Statement

Patients and the public were not involved in this research. Therefore, no ethical permission was required to be obtained.

### Statistical Analysis

The software package STATA (version 14; STATA Inc., College Station, TX, United States) was used for statistical data analysis. Heterogeneity was evaluated using I^2^ index, and *p* < 0.05 was interpreted as heterogeneity. Heterogeneous and non-heterogeneous results were analyzed using the random/fixed effects models and the pooled effect was calculated. Publication bias was assessed using the Harbord test. The DerSimonian and Laird inverse variance method was used to calculate the pooled odds ratio of incidents (OR, 95% CI). In addition, sensitivity analysis was run to investigate the influence of each individual study on the overall meta-analysis summary estimate. The graph of the influence analysis’ result in which the meta-analysis was re-estimated omitting each study in turn was presented. *p* < 0.05 was set as the statistical significance level.

## Results

### Characteristics of Studies

**FIGURE 1:**
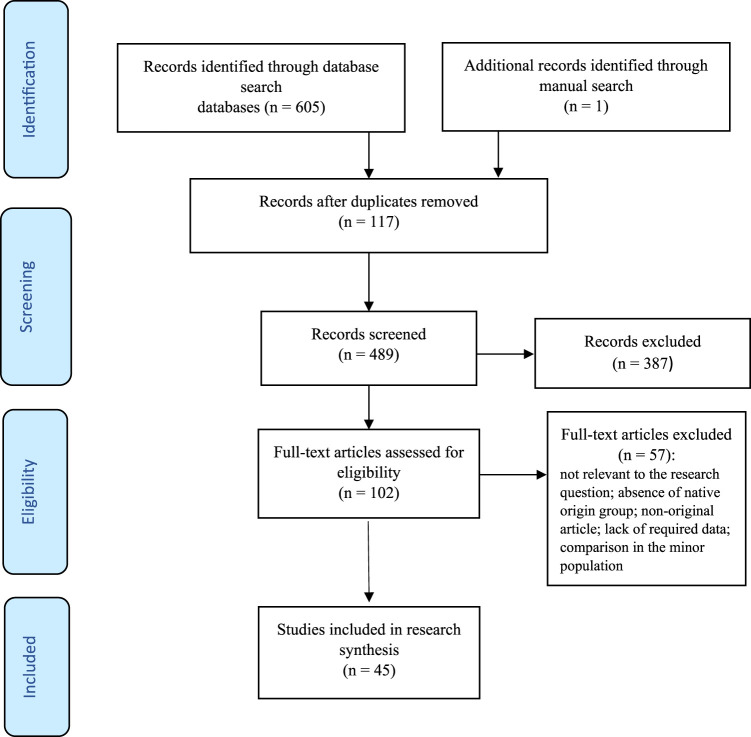
Flowchart of the search process. (Risk of stillbirth, perinatal and neonatal mortality in immigrant women, worldwide, 2021).

The search process yielded 606 studies (Figure 1). A total of 117 duplicate articles were deleted. Using priori selection criteria, 387 studies were excluded based on the screening of titles and abstracts. Therefore, 102 studies were selected for full text appraisal, but 57 studies were further excluded. Finally, 45 studies were included in the systematic review and meta-analysis involving 8,419,435 immigrant women and 40,113,869 native-origin women. The main characteristics of the selected studies have been outlined in Table 1. The studies were conducted in Europe (*n* = 37; Spain [23, 33], Finland [25, 34], Israel [9, 15], Turkey [22, 24, 35–38], Sweden [12, 13, 39, 40], Norway [16, 19, 41–45], Belgium [17, 18, 45, 46], United Kingdom [45, 47], Netherlands [20, 45], Denmark [45, 48], Switzerland [45], Austria [45], Germany [45] and Italy [49]), America (*n* = 8; Canada [21, 26, 50], USA [51–53] and Argentina [4, 54]), Australia (*n* = 5) [8, 10, 11, 14, 55]), and Taiwan (*n* = 1) [56, 57]. The results of quality appraisals of the included studies have been summarized in Supplementary Tables S1, S2. A total of 38 (84%) studies had high [4, 8–19, 21, 23, 25, 26, 33–36, 39–48, 50–53, 55–57], 7 (16%) had moderate quality [20, 22, 24, 37, 38, 49, 54], and no study had low quality.

**TABLE 1:** Baseline characteristic of the studies’ participants. Risk of stillbirth, perinatal and neonatal mortality in immigrant women, worldwide, 2021.

First author, year	Data sources	Year of data collection	Host country	Native origin group sample size	Origin of immigrants	Immigrant group sample size
Auger, 2020	Birth and stillbirth registry	1981–2015	Canada	2,623,245	Arab women	72,677
Barona-Vilar, 2014	Perinatal mortality registry	2005–2008	Spain	162,043	Outside Spain	40,834
Bastola, 2020	Medical birth register of Finland	2004–2014	Finland	350,485	1. Western EU	1. 2,290
					2. Eastern EU	2. 2,566
					3. Russia	3. 11,994
					4. South Asia	4. 1,904
					5. East Asia	5. 4,948
					6. Sub‐Saharan Africa	6. 3,548
					7. Middle East	7. 3,465
					8. Latin America	8. 739
Belihu, 2016	Victorian routine perinatal data registry	1999–2007	Australia	427,755	1. Eritrea	1. 453
					2. Ethiopia	2. 1,094
					3. Somalia	3. 1,861
					4. Sudan	4. 1,404
Burton, 1999	New South Wales midwives data collection	1990–1993	Australia	256,843	Pacific Islands	5,034
Calderon-Margalit, 2015	One medical center	2002–2009	Israel	27,307	Ethiopia	1,319
Çelik, 2019	One hospital	2013–2016	Turkey	48,506	1. Syria	1. 718
					2. Iraq	2. 136
Choi, 2019	Australian national perinatal data collection	2004–2013	Australia	1,735,724	1. Europe	1. 206,999
					2. Middle East and North Africa	2. 69,065
					3. China	3. 47,855
					4. India	4. 48,428
					5. Philippines	5. 25,827
					6. Vietnam	6. 31,729
					7. Asia except middle east	7. 117,417
					8. Latin America	8. 14,475
					9. Africa	9. 39,459
Col Madendag, 2019	One hospital database	2018–2019	Turkey	4,271	Syria	2,040
Dahlen, 2013	New South Wales midwives data collection	2000–2008	Australia	496,668	1. New Zealand	1. 17,293
					2. England	2. 15,218
					3. China	3. 14,526
					4. Vietnam	4. 13,835
					5. Lebanon	5. 12,451
					6. Philippines	6. 9,684
					7. India	7. 8,301
					8. Other	8. 103,761
Ekéus, 2011	Swedish medical birth register, income and population registers	1992–2005	Sweden	1,094,146	1. Western Europe	1. 48,930
					2. Eastern Europe	2. 47,858
					3. Asia	3. 22,748
					4. Latin	4. 13,151
					5. Middle East Asia	5. 65,937
					6. Africa	6. 12,062
					7. Somalia	7. 9,146
Erenel, 2017	One hospital	2013–2016	Turkey	300	Syria	300
Essén, 2000	Malmö database, Swedish medical birth register	1990–1995	Sweden	4,855	All foreign origins	356
Fuster, 2014	Spanish national institute of statistics	2007–2012	Spain	1,874,913	All foreign origins	412,906
Gillet, 2014	Study center for perinatal epidemiology Belgian civil birth registration system	2204–2008	Belgium	261,566	1. Low-income countries	1. 8,066
					2. Middle-income countries	2. 41,985
					3. High-income countries	3. 14,549
Gould, 2003	California linked infant birth/death certificate files	1995–1997	1. USA, white	1. 506,365	1. India	1. 12,899
			2. USA, black	2. 104,888	2. Mexico	2. 433,825
Hsieh, 2011	Birth certificate registration data	1998–2003	Taiwan	1,321,770	Not mentioned	93,161
Johnson, 2005	Birth certificate data	1993–2001	1. USA, black	1. 2,384	Somali	579
			2. USA, white	2. 2,453		
Kanmaz, 2019	One hospital	2013–2016	Turkey	12,198	Syria	4,802
Kiyak, 2020	One hospital	2016–2017	Turkey	940	Syria	616
Liu, 2019	Swedish pregnancy register	2014–2017	Sweden	254,973	Not mentioned	31,897
Liu, 2008	Birth certificates	2002–2007	Taiwan	27,077	1. Mainland China	1. 1,483
					2. Indonesia	2. 1,129
					3. Vietnam	3. 1,081
Lubotzky-Gete, 2017	One hospital	1998–2011	Israel	63,405	1. Ethiopia	1. 1,667
					2. Former Soviet Union	2. 12,920
Madan, 2006	Birth certificates, perinatal mortality data file	1995–2000	US-born white	4,005,671	1. Asian-Indian	1. 76,618
					2. Mexico	2. 1,408,797
Malin, 2009	Finnish medical birth register, statistics Finland	1999–2001	Finland	158,469	1. Nordic	1. 475
					2. Western EU	2. 400
					3. Eastern Europe	3. 597
					4. Former Soviet Union, Russia	4. 1,770
					5. Baltic	5. 496
					6. Middle East, North-Africa	6. 310
					7. South Asia	7. 176
					8. China	8. 135
					9. Iran, Iraq, Afghanistan	9. 428
					10. Southeast Asia	10. 336
					11. Vietnam	11. 302
					12. Africa, excl. Somalia, North Africa	12. 169
					13. Somalia	13. 817
					14. Latin America and Caribbean	14. 121
Mozooni, 2020	Perinatal, birth, death, hospital, birth defects registrations	2005–2013	Australia	172,571	1. White	1. 48,546
					2. Asia	2. 18,212
					3. India	3. 5,503
					4. Africa	4. 4,155
					5. Māori	5. 2,941
					6. Other	6. 9,038
Naimy, 2013	Medical birth registry of Norway, Norwegian central person registry	1986–2005	Norway	1062744	1. Pakistan	1. 11,351
					2. Vietnam	2. 6,169
					3. Somalia	3. 5,410
					4. Sri Lanka	4. 4,933
					5. Philippines	5. 4,662
					6. Iraq	6. 3,829
					7. Thailand	7. 3,204
					8. Afghanistan	8. 665
Opondo, 2020	Statutory birth and death registration data for England and Wales, National Health Service Numbers for Babies (NN4B) birth notifications system, office for national statistics	2006–2012	United Kingdom	3,009,231	1. White (other)	1. 340,526
					2. India	2. 132,651
					3. Pakistan	3. 180,651
					4. Bangladesh	4. 62,948
					5. Black Caribbean	5. 47,505
					6. Black Africa	6. 154,076
					7. Mixed or other	7. 419,970
					8. Not mentioned	8. 287,756
Ozel, 2018	One hospital	2015	Turkey	576	Syria	576
Racape, 2013	Birth and death certificates	1998–2008	Belgium	39,893	1. Morocco	1. 12,371
					2. Moroccan naturalised	2. 15,108
					3. Sub-Sahara	3. 6,322
					4. Sub-Saharan naturalised	4. 3,070
					5. Turkey	5. 3,185
					6. Turkey naturalised	6. 3,673
Racape, 2016	Birth and death certificates	1998–2010	Belgium	1,029,471	1. Western Europe	1. 98,189
					2. Western Europe naturalized Belgian	2. 34,701
					3. Turkey	3. 20,451
					4. Turkey naturalized Belgian	4. 21,878
					5. Maghreb	5. 51,224
					6. Maghreb naturalized Belgian	6. 46,681
					7. Sub-Saharan Africa	7. 26,621
					8. Sub-Saharan Africa naturalized Belgian	8. 11,420
					9. Eastern Europe	9. 17,420
					10. Eastern Europe naturalized Belgian	10. 5,412
Raimondi, 2013	Public maternity hospital	2009	Argentine	1,000	Not mentioned	1,000
Råssjö, 2013	Records of antenatal and obstetric care	2001–2009	Sweden	513	Somali	258
Sørbye, 2014	Statistic Norway, medical birth registry of Norway	1995–2010	Norway	712,430	Pakistan	10,615
Vang, 2016	Birth-infant death records	1990–2005	Canada	2,856,394	1. United States	1. 41,601
					2. North Africa	2. 17,991
					3. Sub-Saharan Africa	3. 24,339
					4. Haiti	4. 20,057
					5. Non-Spanish Caribbean	5. 10,499
					6. Latin America	6. 38,702
					7. Pakistan	7. 7,500
					8. South Asia	8. 68,558
					9. Central/West Asia	9. 27,491
					10. East/SE Asia	10. 138,024
					11. Europe	11. 108,515
					12. Rest of the world	12. 10,970
Vangen, 2002	Medical birth registry of Norway	1986–1998	Norway	702,192	Somalia	1,733
Vangen, 2000	Medical birth registry of Norway	1986–19958	Norway	535,600	1. Turkey, Morocco	1. 2,758
					2. Pakistan	2. 4,929
					3. India, Sri Lanka	3. 2,643
					4. Vietnam	4. 2,704
					5. Philippines	5. 1,985
					6. Horn of Africa	6. 1,406
					7. Chile Brazil	7. 1,466
Verschuuren, 2020	One midwifery practice, hospital databases	2012–2016	Netherlands	2,323	1. Eritrea	1. 65
					2. Syria	2. 75
					3. Middle east	3. 75
					4. Sub-Saharan Africa	4. 50
					5. Eastern Europe	5. 43
					6. Other	6. 18
Vetter, 2013	12 public hospitals	2008	Argentine	9,155	Not mentioned	1,715
Vik, 2019	Medical birth registry of Norway and statistics Norway	1990–2013	Norway	1,136,637	Central Europe, Eastern Europe, Central Asia, Latin America, Caribbean, North Africa, Middle East, South Asia, Sub-Saharan Africa, Southeast Asia, East Asia, Oceania, other	195,725
Vik, 2020	Medical birth registry of Norway and statistics Norway	1990–2016	Norway	66,006	Not mentioned	30,062
Villadsen, 2010	Birth registries or surveys	1990–2005	1. Belgium	1. 238,233	Turkey	8,717
			2. Denmark	2. 812,305		
			3. Norway	3. 675,387		
			4. Sweden	4. 1,344,237		
			5. Switzerland	5. 900,875		
			6. Austria	6. 408,695		
			7. United Kingdom	7. 1,037,348		
			8. Germany	8. 1,296,798		
			9. Netherland	9. 935,858		
Villadsen, 2009	Danish medical birth registry, causes of death registry and socioeconomic and demographic data	1981–2003	Denmark	1,278,539	1. Turkey	1. 22,717
					2. Lebanon	2. 9,280
					3. Pakistan	3. 8,481
					4. Former Yugoslavia	4. 8,020
					5. Somali	5. 6,415
Wanigaratne, 2018	IRCC-PRD, Ontario’s healthcare registry, abstract database, general’s vital statistics- death registry	2002–2014	Canada	29,023	Not mentioned	29,023
Zanconato, 2011	One public hospital	2005–2009	Italy	6,365	1. Central and Eastern Europe	1. 1,001
					2. South and East Asia	2. 539
					3. Middle East and North Africa	3. 460
					4. Sub-Saharan Africa	4. 448
					5. Central and South America	5. 213

### Meta-Analysis of Outcomes

**FIGURE 2:**
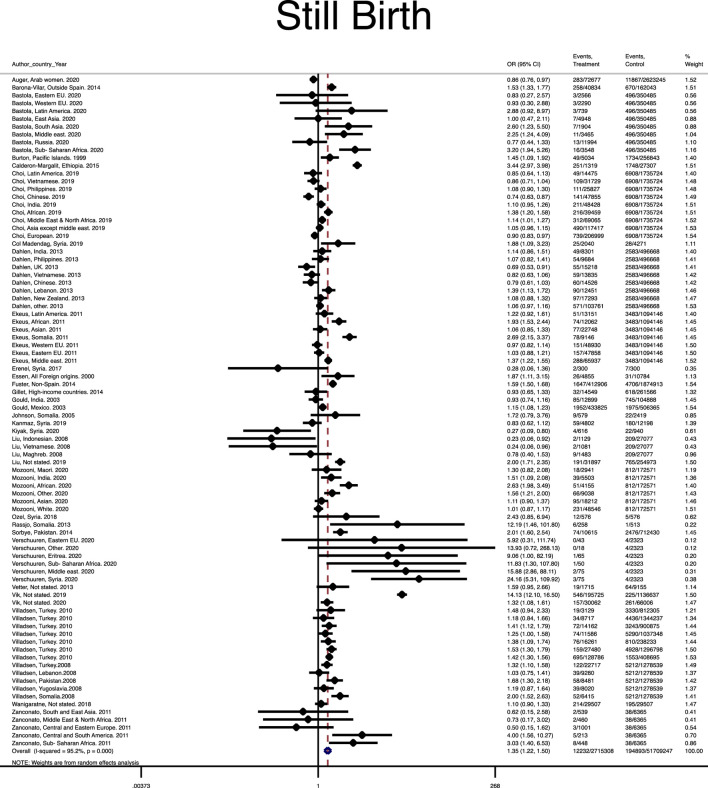
Forest plot of the pooled odds ratio of stillbirth. (Risk of stillbirth, perinatal and neonatal mortality in immigrant women, worldwide, 2021).

A total of 31 studies [4, 8–13, 20–23, 26, 33–41, 43–46, 48, 49, 51, 52, 55, 57] reported stillbirth in 12,232 out of 2,715,308 immigrant women and 72,100 out of 19,262,686 native-origin women. Although there were statistical significant heterogenicities in the studies (I-squared = 95.5%), no publication bias was found (Harbord test *p* value = 0.160). The pooled risk of stillbirth among immigrant women was 1.35 folds higher than that of native-origin women (Pooled OR = 1.35, 95% CI = 1.22–1.50) (Figure 2).

**FIGURE 3:**
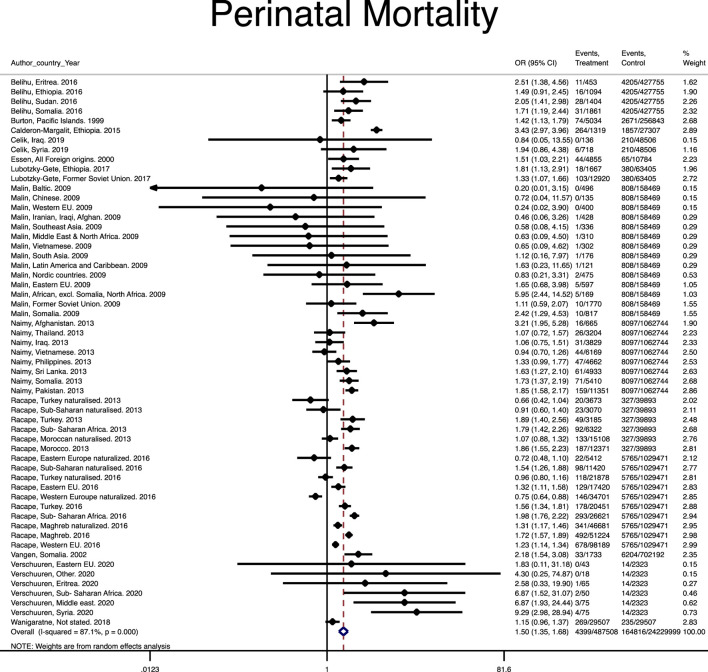
Forest plot of the pooled odds ratio of perinatal mortality. (Risk of stillbirth, perinatal and neonatal mortality in immigrant women, worldwide, 2021).

A total of 17 studies [4, 8, 10, 11, 26, 33, 34, 42, 43, 46, 47, 50–54, 56] reported the perinatal mortality among 4,399 out of 487,508 immigrant women and 30,838 out of 3,859,199 native-origin women. However, heterogeneity (I-squared = 87.1%) without publication bias (Harbord test *p* value = 0.340) was found across the studies. The pooled risk of perinatal mortality among immigrant women was significantly 1.5 folds higher than native-origin women (Pooled OR = 1.50, 95% CI = 1.35–1.68) (Figure 3).

**FIGURE 4:**
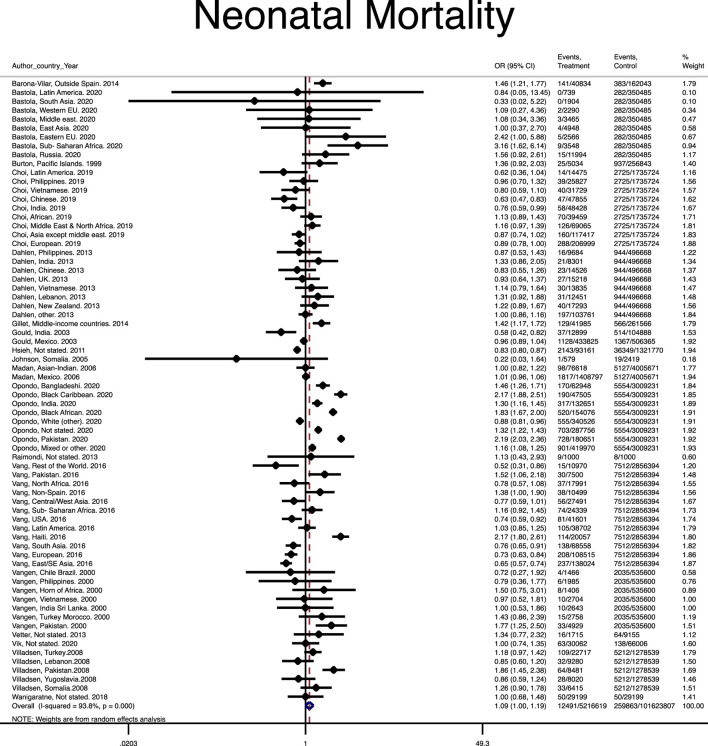
Forest plot of the pooled odds ratio of neonatal mortality. (Risk of stillbirth, perinatal and neonatal mortality in immigrant women, worldwide, 2021).

A total of 14 studies [8, 9, 13–20, 24–26, 48] reported neonatal mortality among 12,491 out of 5,216,619 immigrant women and 70,464 out of 16,991,984 native-origin women. Statistically significant heterogenicities (I-squared = 93.6%), without publication bias were found (Harbord test *p* value = 0.211) in the studies. Although immigrant women faced a greater risk of neonatal morbidity compared to native-origin women, its risk was not statistically significant (Pooled OR = 1.09, 95% CI = 1.00–1.19) (Figure 4).

### Sensitivity Analysis

Sensitivity analysis was performed to show the influence of each individual study on the overall meta-analyses’ summary estimates. All results were highly consistent with the main data analysis’ results and no substantial modification of the estimates of change was found after the exclusion of any individual study (Supplementary Figures S1A–C).

## Discussion

The results of this systematic review and meta-analysis revealed that the risk of stillbirth, perinatal and neonatal mortality increased among immigrant women compare to native-origin women in host countries.

Despite advances in antenatal and intrapartum care, perinatal mortality continues to be a major burden on the healthcare system. It can have devastating impacts on parents because of its unexpected nature [58, 59]. Adverse outcomes are multifactorial with a number of issues appearing to coalesce to immigrant women. Our review showed that immigrant women represented a high-risk group for stillbirth and perinatal mortality, which needed special care and attention during pregnancy and childbirth. Maternal background factors cause poorer outcomes in immigrant women. These women have medical problems such as infectious diseases or female genital mutilation leading to complications for themselves and their newborns [60–62]. Immigrant women have a poor access to maternity and public healthcare services in host countries [63–65], which can increase the risk of mortality among their newborns due to maternal disorders, unintended pregnancy, grand multiparity and teenage pregnancies [66, 67], preterm birth [68, 69], and undiagnosed congenital fetal anomalies [70, 71].

Health immigration policies of host countries and the immigrant legal status appear to influence their access to maternity health services. Those women without a legal resident permit are most vulnerable [67]. Gieles et al (2019) in a systematic review stated that positive integration policies without discrimination and adoption to the host-country nationality decreased perinatal mortality and morbidity among immigrant women and their newborns [69]. Additionally, late booking and delayed utilization of prenatal care with the presence of cultural and language barriers hinder appropriate access to healthcare services by immigrant women leading to higher mortality among their newborns compared to native-origin women [63]. Despite free access to maternity care in host countries, immigrant women usually utilize less antenatal care [72]. Poor communication between these women and healthcare providers in host countries such as unsupported and fearful interactions, being rude, discriminatory or insensitive to their specific needs can lead to late booking or delay in the use of maternity care services, which have negative impacts on prenatal care utilization [63, 73]. Cultural issues regarding prenatal or antenatal care as the facet of medicalization of childbirth care among immigrant women and different understanding of health and diseases hinder appropriate healthcare interventions in delivery, which are accompanied with higher stillbirth or perinatal mortality incidents in immigrant women [62].

It has been well documented that socio-economically vulnerable populations exhibit a higher mortality rate [74]. Immigrant women also experience a larger burden of poverty-related adverse circumstances such as the low social level, unhealthy lifestyle and behavior, malnutrition, which can play significant roles in poorer mortality outcomes among these women [73–75]. Consistently, perinatal mortality in immigrants from low-income settings of conflicting countries and having economic hardship generally occur more frequently that in the host population [67].

Although it is not most certainly observed in every immigrant group in every host nation, immigrants have often lower education levels and are more often single parents. Therefore, they have the risk factors of perinatal morbidity and stillbirth [76].

Studies mostly show that immigrant women are relatively younger than native-origin women [77–79], which may support the “healthy migrant” hypothesis, suggesting that the health of immigrants prior to immigration may be better than the health of the general population in both the sending and the receiving countries [80]. However, information on the higher rate of perinatal and neonatal mortality and stillbirth among immigrant women can be translated to health disparities that need more attention by healthcare organizations, regulatory bodies, educational institutions, and the society.

The results of our review should be interpreted with caution. Given the lack of data in the included studies, we could not adjust the legal status, reason for immigration, and length of the residence of immigrant women as well as background factors of immigrants and native-origin populations. Also, most data used in this review were based on population and health-related registers, which typically lacked detailed background data on women and their newborns. The frequent movement of immigrants usually leads to less complete registrations of their health status.

In conclusion, our review showed that immigrant women and their newborns had the higher risk of stillbirth, and perinatal and neonatal mortality compared to native-origin women. The result of sensitivity analyses supported the stability and robustness of our review results. Healthcare providers and policy makers should improve the provision of maternal and neonatal healthcare for the immigrant population.
