# Supplementary material for: A Systematic Review and Meta-Analysis of the Risk of Stillbirth, Perinatal and Neonatal Mortality in Immigrant Women
Source: Int J Public Health. 2022 May 18;67:1604479. doi: 10.3389/ijph.2022.1604479 (PMC9156626; doi:10.3389/ijph.2022.1604479)
Supplement: Supplementary file 1 [file DataSheet1.docx]

**Supplementary file**

Supplementary table 1: Quality assessment of the selected studies using the Newcastle–Ottawa Quality Assessment Scale for cohort studies. Risk of stillbirth, perinatal and neonatal mortality in immigrant women, worldwide, 2021.

|  | **SELECTION** | | | | **COMPARABILITY** | **Outcome** | | | **Total scores** |
| --- | --- | --- | --- | --- | --- | --- | --- | --- | --- |
| **Author, Year** | Representativeness of the exposed cohort * | Selection of the non-exposed cohort * | Ascertainment of exposure * | No outcome of interest at the start of the study * | A: Study controls for age and/or BMI *  B: Study controls for other confounders * | A: doctor’s diagnosis OR objective measurements *  B: parent/self-reported doctor’s diagnosis OR the use of medication * | Follow-up long enough for outcomes * | Adequacy of the follow up of cohorts * |  |
| Auger et al, 2020 | * | * | * | * | ** | * | * | * | 9* |
| Burton, 1999 | * | * | * | * | ** | * | * | * | 9* |
| ÇELIK et al, 2019 |  |  | * | * |  | * | * | * | 5* |
| Choi et al, 2019 | * | * | * | * | ** | * | * | * | 9* |
| Col Madendag et al, 2019 |  |  | * | * | ** | * | * | * | 7* |
| Ekeus et al, 2011 | * | * | * | * | ** | * | * | * | 9* |
| Essen et al, 2000 | * | * | * | * | ** | * | * | * | 9* |
| Johnson et al, 2005 | * | * | * | * | ** | * | * | * | 9* |
| Kanmaz et al, 2019 |  |  | * | * | ** | * | * | * | 7* |
| Kiyak et al, 2020 |  |  | * | * |  | * | * | * | 5* |
| Liu et al, 2019 | * | * | * | * | ** | * | * | * | 9* |
| Lubotzky-Gete et al, 2017 |  |  | * | * | ** | * | * | * | 7* |
| Mozooni et al, 2020 | * | * | * | * | ** | * | * | * | 9* |
| Ozel et al, 2018 |  |  |  | * | ** | * | * | * | 6* |
| Racape et al, 2013 | * | * | * | * | ** | * | * | * | 9* |
| Raimondi et al, 2013 |  |  | * | * |  | * | * | * | 5* |
| Råssjö et al, 2013 | * | * | * | * |  | * | * | * | 7* |
| Wanigaratne et al, 2018 | * | * | * | * | ** | * | * | * | 9* |
| Zanconato et al, 2011 |  |  | * | * | * | * | * | * | 6* |

Supplementary table 2. Quality assessment of the selected studies using the Newcastle–Ottawa Quality Assessment Scale for cross-sectional studies. Risk of stillbirth, perinatal and neonatal mortality in immigrant women, worldwide, 2021.

|  | **SELECTION** | | | | **COMPARABILITY** | **Outcome** | | **Total scores** |
| --- | --- | --- | --- | --- | --- | --- | --- | --- |
| **Author** | Representativeness of the samples | Sample size | Non-responders | Ascertainment of the exposure | A: study controls for age and/or BMI  B: control for any additional factor | Assessment of the outcome  a) Independent blind assessment  b) Record linkage  c) Self report | Statistical test |  |
| Barona-Vilar et al, 2014 | * | * | * | * | * | ** | * | 8* |
| Bastola et al, 2020 | * | * | * | * | ** | ** | * | 9* |
| Belihu et al, 2016 | * | * | * | * | ** | ** | * | 9* |
| Calderon-Margalit et al, 2015 |  | * | * | * | ** | ** | * | 7* |
| Dahlen et al, 2013 | * | * | * | * | ** | ** | * | 9* |
| Erenel et al, 2017 |  |  |  | * | ** | ** | * | 6* |
| Fuster et al, 2014 | * | * | * | * | ** | ** | * | 9* |
| Gillet et al, 2014 | * | * | * | * | ** | ** | * | 9* |
| Gould et al, 2003 | * | * | * | * |  | ** | * | 7* |
| Hsieh et al, 2011 | * | * | * | * | ** | ** | * | 9* |
| Liu et al, 2008 | * | * | * | * | ** | ** | * | 9* |
| Madan et al, 2006 | * | * | * | * |  | ** | * | 7* |
| Malin and Gissler, 2009 | * | * | * | * | ** | ** | * | 9* |
| Naimy et al, 2013 | * | * | * | * | ** | ** | * | 9* |
| Opondo et al, 2020 | * | * | * | * | ** | ** | * | 9* |
| Racape et al, 2016 | * | * | * | * | ** | ** | * | 9* |
| Sørbye et al, 2014 | * | * | * | * | ** | ** | * | 9* |
| Vang, 2016 | * | * | * | * | ** | ** | * | 9* |
| Vangen et al, 2002 | * | * | * | * | ** | ** | * | 9* |
| Vangen et al, 2008 | * | * | * | * | ** | ** | * | 9* |
| Verschuuren et al, 2020 |  |  | * | * |  | ** | * | 5* |
| Vetter et al, 2013 | * | * | * | * | ** | ** | * | 9* |
| Vik et al, 2019 | * | * | * | * | ** | ** | * | 9* |
| Vik et al, 2020 | * | * | * | * | ** | ** | * | 9* |
| Villadsen, 2009 | * | * | * | * | ** | ** | * | 9* |
| Villadsen et al, 2010 | * | * | * | * | * | ** | * | 8* |

Supplementary Figure 1. Plots of sensitivity analysis of results (A) still birth (B) neonatal mortality and (C) perinatal mortality. Risk of stillbirth, perinatal and neonatal mortality in immigrant women, worldwide, 2021.

(A) Still birth

(B) Neonatal mortality

(C) Perinatal mortality
